# Sarcopenia and anti‐seizure medication response in juvenile myoclonic epilepsy

**DOI:** 10.1002/brb3.3464

**Published:** 2024-03-11

**Authors:** Jinseung Kim, Ho‐Joon Lee, Dong Ah Lee, Kang Min Park

**Affiliations:** ^1^ Department of Family Medicine Busan Paik Hospital Inje University College of Medicine Busan Republic of Korea; ^2^ Department of Radiology Haeundae Paik Hospital Inje University College of Medicine Busan Republic of Korea; ^3^ Department of Neurology Haeundae Paik Hospital Inje University College of Medicine Busan Republic of Korea

**Keywords:** epilepsy, magnetic resonance imaging, sarcopenia

## Abstract

**Introduction:**

This study aimed to investigate the presence of sarcopenia in patients with juvenile myoclonic epilepsy (JME) and the association between sarcopenia and response to anti‐seizure medication (ASM) in patients with JME.

**Methods:**

We enrolled 42 patients with JME and 42 healthy controls who underwent brain magnetic resonance imaging with three‐dimensional T1‐weighted imaging. We measured the temporal muscle thickness (TMT), a radiographic marker for sarcopenia, using T1‐weighted imaging. We compared the TMT between patients with JME and healthy controls and analyzed it according to the ASM response in patients with JME. We also performed a receiver operating characteristic (ROC) curve analysis to evaluate how well the TMT differentiated the groups.

**Results:**

The TMT in patients with JME did not differ from that in healthy controls (9.630 vs. 9.956 mm, *p *= .306); however, ASM poor responders had a lower TMT than ASM good responders (9.109 vs. 10.104 mm, *p *= .023). ROC curve analysis revealed that the TMT exhibited a poor performance in differentiating patients with JME from healthy controls, with an area under the ROC curve of .570 (*p *= .270), but good performance in differentiating between ASM good and poor responders, with an area under the ROC curve of .700 (*p *= .015).

**Conclusion:**

The TMT did not differ between patients with JME and healthy controls; however, it was reduced in ASM poor responders compared to ASM good responders, suggesting a link between ASM response and sarcopenia in patients with JME. TMT can be used to investigate sarcopenia in various neurological disorders.

## Graphical abstract

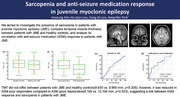

## 1 INTRODUCTION

Juvenile myoclonic epilepsy (JME) is an epileptic syndrome that causes idiopathic generalized epilepsy along with childhood absence epilepsy, juvenile absence epilepsy, and epilepsy with generalized tonic–clonic seizures alone (Hirsch et al., 2022). It is the most common type of idiopathic generalized epilepsy, accounting for approximately 9.3% of all epilepsies (Syvertsen et al., 2017).

Sarcopenia, introduced in 1989 by Irwin Rosenberg, refers to the age‐related loss of muscle mass, strength, and function and is regarded as a natural part of the aging process (“Epidemiologic and methodologic problems in determining nutritional status of older persons. Proceedings of a conference. Albuquerque, New Mexico, October 19–21, 1988,” 1989). It could have significant implications for an individual's overall health and quality of life. In a meta‐analysis of studies published in 2022, the occurrence of sarcopenia ranged from 10% to 27%, with the occurrence of severe sarcopenia falling within a range of 2%–9% (Petermann‐Rocha et al., 2022). Both primary sarcopenia associated with aging as well as secondary sarcopenia resulting from other medical conditions, including neurological disorders, typically begin to occur in middle age and progress thereafter (Cruz‐Jentoft & Sayer, 2019; Yang et al., 2022). Evidence suggests that sarcopenia can co‐occur in various neurological diseases, such as Parkinson's disease, multiple sclerosis, stroke, dementia, and myasthenia gravis (Cai et al., 2021; Cho et al., 2022; Ryan et al., 2017; Yang et al., 2022). However, research regarding the relationship between sarcopenia and epilepsy is lacking. Dysfunction in mitochondria, neuromuscular signaling, endocrine factors, and inflammation play a significant role in the development of sarcopenia, which is more common in patients with epilepsy than in healthy individuals (Bakhtadze et al., 2021; Cruz‐Jentoft & Sayer, 2019; McNamara et al., 2017; Waldbaum & Patel, 2010; Yang et al., 2022). In addition, sleep disturbance is prevalent among patients with epilepsy, which is also considered one of the contributing factors to sarcopenia (Koike et al., 2023; Smith et al., 2022). Therefore, we would expect sarcopenia to occur more commonly in patients with epilepsy, including JME.

There are several ways to diagnose sarcopenia, such as physical examination to assess muscle mass and grip strength; functional tests, including gait speed, chair stand, and balance assessments; and muscle mass measurement using dual‐energy X‐ray absorptiometry or computed tomography (Jang, 2018; Lin et al., 2019). In addition, measurement of temporal muscle thickness (TMT) is a recently introduced method to evaluate sarcopenia. This attractive method has the advantage of using previously obtained brain magnetic resonance imaging (MRI) data. Previous research has shown that the TMT correlates well with hand grip strength and psoas muscle area measurements, which are well‐known markers of sarcopenia (Ranganathan et al., 2014; Steindl et al., 2020). The TMT has been used to study sarcopenia in patients with brain tumors, which shows that the degree of sarcopenia is associated with poor prognosis (Huq et al., 2021).

Recently, sarcopenia has been reported to be related to polypharmacy (Pana et al., 2022; Prokopidis et al., 2023). Especially both low muscle mass and low muscle attenuation have been associated with poor tolerance to chemotherapy in patients with cancer (Ryan et al., 2019). However, no studies have been conducted on the relationship between drug response and sarcopenia in patients with epilepsy.

The aims of this study were to investigate (1) the presence of sarcopenia in patients with JME using the TMT and (2) the association between sarcopenia and anti‐seizure medication (ASM) response in patients with JME. We hypothesized that (1) sarcopenia and JME could coexist, and (2) there is a clear association between ASM response and sarcopenia in patients with JME.

## 2 METHODS

### 2.1 Participants

This study was approved by the institutional regional board of our institute. We retrospectively enrolled 42 patients diagnosed with JME, adhering to the following inclusion criteria: (1) patients who were newly diagnosed with JME at our hospital and exhibited clinical and electroencephalography findings consistent with JME; (2) patients who underwent three‐dimensional (3D) T1‐weighted imaging with high‐quality data available for quantitative analysis at the time of their JME diagnosis; (3) patients who had no structural abnormalities on brain MRI; and (4) patients who did not have any other medical, neurological, or psychiatric disorders apart from JME. We collected data on the clinical characteristics of the patients with JME, including age, sex, age at seizure onset, duration of epilepsy (measured from the first seizure to the time of MRI acquisition), and seizure type. We divided patients with JME into two groups: ASM good responders and ASM poor responders. In cases where patients respond well to the first ASM and maintain seizure‐free status during the follow‐up period, they were classified as ASM good responders. Conversely, when the efficacy of the first ASM was insufficient and additional ASMs were required, they were categorized as ASM poor responders.

Additionally, we enrolled a control group of 42 age‐ and sex‐matched healthy individuals. These individuals had previously been enrolled as part of a normal control group for research purposes in a prior study (Jang et al., 2017). They exhibited normal brain MRI scans and did not present with any other medical, neurological, or psychiatric disorders.

### 2.2 MRI acquisition

Both patients with JME and healthy controls underwent brain MRI using identical sequences. The scans were performed using a three‐tesla MRI scanner equipped with a 32‐channel head coil (Achieva Tx; Phillips Healthcare). The MRI protocol included three key sequences: 3D fluid‐attenuated inversion recovery, coronal T2‐weighted imaging, and 3D T1‐weighted imaging. These sequences constitute standard MRI protocols routinely employed for patients with JME at our hospital. The 3D T1‐weighted was acquired in the sagittal plane, using a turbo‐field echo sequence with the following parameters: TI = 1300 ms, repetition time/echo time = 8.6/3.96 ms, flip angle = 8°, and isotropic voxel size = 1 mm^3^.

### 2.3 TMT measurement

**FIGURE 1:**
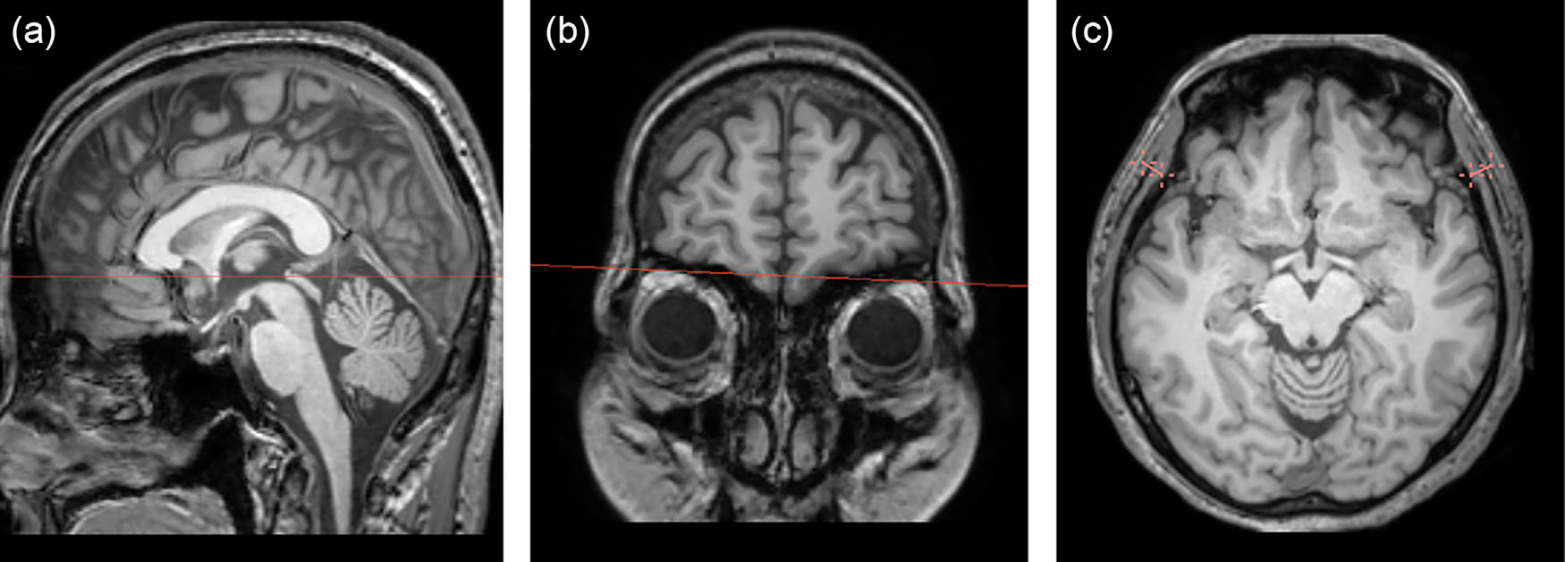
Illustration of the temporalis muscle thickness measurement procedure: (A) images are reformatted to the axial plane parallel to the anterior commissure‐posterior commissure line; (B) the view is navigated to the orbital roof level; (C) thickness measurements of the temporalis muscle are taken on both sides, with the sylvian fissure used as an anterior–posterior reference point.

A board‐certified radiologist (H.J.L., with 9 years of subspecialty experience in neuroradiology) measured the TMT on the right and left sides on 3D T1‐WI. The images were reformatted to an axial plane parallel to the anterior commissure‐posterior commissure line. The TMT was measured perpendicular to the long axis of the temporalis muscle, using the orbital roof and sylvian fissure as landmarks. Image reformatting and measurements were performed using 3D Slicer (version 5.4.0, https://www.slicer.org) (Fedorov et al., 2012; Kikinis & Pieper, 2011). The measurements for each side were averaged and used for further analysis. Figure 1 illustrates the TMT measurement procedure.

### 2.4 Statistical analysis

We employed the independent samples *t*‐test to compare age and TMT and used the Mann–Whitney test to compare the age at seizure onset and duration of epilepsy between the groups. The chi‐square test was used to analyze sex and seizure type differences between the groups. We compared the TMT between patients with JME and healthy controls and those with good and poor ASM response. We also performed receiver operating characteristic (ROC) curve analysis to evaluate how well TMT differentiated patients with JME from healthy controls and good and poor ASM responders. For correlation analysis between the TMT and clinical factors, we employed the Pearson correlation test. All statistical analyses were executed using MedCalc software (version 20.014, MedCalc Software, Ostend, Belgium; accessible at https://www.medcalc.org; 2021). Statistical significance was set at a threshold of *p* < .05.

## 3 RESULTS

### 3.1 Clinical characteristics of participants

There were no significant differences in age (25.8 vs. 25.0 years, *p *= .607) or sex distribution (61% vs. 61% males, *p *= 1.000) between the JME and healthy control groups. Table 1 shows the detailed clinical characteristics of patients with JME. Of the 42 patients with JME, 22 and 20 were ASM good and poor responders, respectively. There were no differences in age, sex, age at seizure onset, duration of epilepsy, or seizure type between the groups.

**TABLE 1:** Clinical characteristics of patients with juvenile myoclonic epilepsy and healthy controls.

	Patients with JME (*N* = 42)	Healthy controls (*N* = 42)	*p*‐Value
Age, years	25.8 ± 8.6	25.0 ± 4.2	.607
Male, *n* (%)	26 (61.9)	26 (61.9)	1.000
Age at seizure onset, years	16.2 (11.5–18.7)		
Duration of epilepsy, months	84 (31–135)		
Seizure types			
Myoclonic seizures, *n* (%)	42 (100)		
GTC seizures, *n* (%)	34 (80.9)		
Absence seizures, *n* (%)	14 (33.3)		
	ASM good responders (*N* = 22)	ASM poor responders (*N* = 20)	*p*‐Value
Age, years	26.4 ± 5.6	25.0 ± 9.1	.547
Male, *n* (%)	16 (72.7)	10 (50.0)	.134
Age at seizure onset, years	15.3 (11.0–19.7)	16.8 (12.1–18.3)	.715
Duration of epilepsy, months	88 (41–139)	80 (24–101)	.548
Seizure types			
Myoclonic seizures, *n* (%)	22 (100)	20 (100)	1.000
GTC seizures, *n* (%)	19 (86.3)	15 (75.0)	.354
Absence seizures, *n* (%)	8 (36.3)	6 (30.0)	.666

Abbreviattions: ASM, anti‐seizure medication; GTC, generalized tonic–clonic; JME, juvenile myoclonic epilepsy.

### 3.2 Difference in TMT between JME patients and healthy controls

**FIGURE 2:**
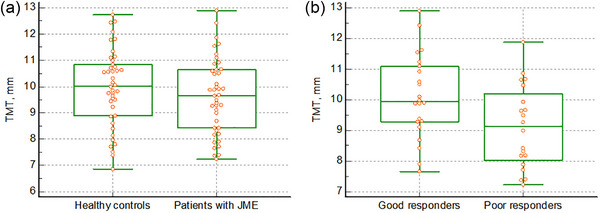
Difference in temporal muscle thickness between juvenile myoclonic epilepsy and healthy controls The temporal muscle thickness in patients with juvenile myoclonic epilepsy was not significantly different compared to healthy controls (9.630 vs. 9.956 mm, *p *= .306) (A), but poor responders to anti‐seizure medication had reduced temporal muscle thickness compared to good responders to anti‐seizure medication (9.109 vs. 10.104 mm, *p *= .023) (B). JME, juvenile myoclonic epilepsy; TMT, temporal muscle thickness.

The TMT in patients with JME was not significantly different compared to healthy controls (9.630 ± 1.436vs. 9.956 ± 1.470 mm, *p *= .306). However, ASM poor responders had a lower TMT than ASM good responders (9.109 ± 1.349 vs. 10.104 ± 1.373 mm, *p *= .023) (Figure 2).

### 3.3 ROC curve analysis

**FIGURE 3:**
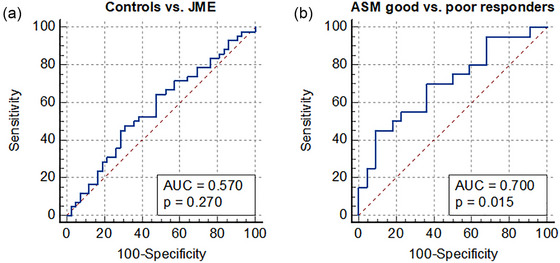
Receiver operating characteristic (ROC) curve analysis ROC curve analysis revealed that temporal muscle thickness exhibited poor performance in differentiating patients with juvenile myoclonic epilepsy from healthy controls, with an area under the ROC curve of .570 (*p *= .270) (A), but good performance in differentiating between anti‐seizure medication good and poor responders, with an area under the ROC curve of .700 (*p *= .015) (B). AUC, area under the receiver operating characteristic curve.

ROC curve analysis revealed that the TMT exhibited poor performance in differentiating patients with JME from healthy controls, with an area under the ROC curve (AUC) of .570 (*p *= .270), but good performance in differentiating between ASM good and poor responders, with an AUC of .700 (*p *= .015) (Figure 3).

### 3.4 Correlation analysis

The TMT was not correlated with any clinical characteristics in patients with JME, including age (*r* = .125, *p *= .428), age at seizure onset (*r* = .009, *p *= .954), or duration of epilepsy (*r* = .093, *p *= .556).

## 4 DISCUSSION

To our knowledge, this is the first study to evaluate sarcopenia in patients with epilepsy. In this study, we found that the TMT in patients with JME was not significantly different compared to healthy controls, suggesting that sarcopenia was not more common in patients with JME than in healthy controls. However, poor ASM responders had a lower TMT than good ASM responders, suggesting an association between ASM response and sarcopenia in patients with JME. In addition, our study demonstrated that the TMT could exhibit poor performance in differentiating patients with JME from healthy controls but good performance in differentiating between ASM good and poor responders.

Sarcopenia is a progressive and generalized skeletal muscle disorder commonly occurring in the elderly population and reduces the overall health and quality of life. However, recent studies have shown that sarcopenia occurs not only in older adults but also in patients with various neurological diseases. Therefore, this study investigated the association between sarcopenia and epilepsy. As epilepsy has a diverse spectrum, this study was limited to a single epilepsy syndrome, JME. However, contrary to our expectations, we did not observe any association between sarcopenia and JME. Several plausible explanations exist for this observation. First, the mean age of patients with JME in this study was relatively young (25 years). Although sarcopenia has been found to accompany several neurological diseases, it is mainly associated with diseases that occur in older patients, such as Parkinson's disease, Alzheimer's disease, or ischemic stroke (Cai et al., 2021; Cho et al., 2022; Ryan et al., 2017; Yang et al., 2022). Thus, different results could have been obtained if the study included elderly rather than young epilepsy patients. Second, we used TMT measurement to investigate sarcopenia in patients with JME. TMT is a known biomarker for sarcopenia and is well correlated with hand grip strength and psoas muscle area measurements (Ranganathan et al., 2014; Steindl et al., 2020). However, the diagnosis of sarcopenia has recently been changed to include not only the detection of low muscle mass but also decreased muscle function and low physical performance (Cruz‐Jentoft et al., 2019). Because we did not directly measure muscle function and physical performance in patients with JME and healthy controls, it was difficult to rule out the possibility that this negative result originated from the measurement methods.

However, this study revealed a significant association between sarcopenia and the ASM response in patients with JME. ASM poor responders had a lower TMT and, therefore, were more likely to have sarcopenia than ASM good responders. Because this was the first study to investigate the association between ASM response and sarcopenia in patients with epilepsy, the exact cause was unknown. However, it could be multifaceted, and several possibilities can be considered. First, ASM response can be related to the nature of epileptogenesis, which is achieved through a complex interaction of various factors, including mitochondrial pathology, inflammation, and genetic susceptibility (Singh & Singh, 2021). This complex interaction may cause predispositions for sarcopenia. Both inflammation and mitochondrial pathology are commonly cited as a cause of epileptogenesis, which are also related with sarcopenia (Singh & Singh, 2021; Xu & Wen, 2023). The association between mitochondrial dysfunction and epileptogenesis revolves around the intricate interplay between cellular energy metabolism and neuronal excitability. Mitochondria, known as the powerhouse of the cell, plays a crucial role in generating adenosine triphosphate, the primary energy source for cellular processes, including neuronal activity. Dysfunction in mitochondrial function can disrupt adenosine triphosphate production, leading to energy deficits and impaired cellular homeostasis, which may contribute to neuronal hyperexcitability and seizure susceptibility (Khurana et al., 2013). In addition, mitochondrial dysfunction with impaired protein homeostasis, bioenergetic failure, oxidative stress, unbalanced of dynamics, impaired autophagy, and impaired biogenesis produce reduced energy production, mitophagy, and cell apoptosis, resulting to sarcopenia (Xu & Wen, 2023). Furthermore, inflammation can induce structural and functional changes in the brain, including synaptic reorganization, neuronal loss, and gliosis, which can perpetuate epileptic activity and contribute to the development of drug‐resistant epilepsy. Inflammatory processes may also interact with other pathogenic mechanisms implicated in epileptogenesis, such as oxidative stress, mitochondrial dysfunction, and alterations in ion channel function, creating a complex network of interconnected pathways underlying seizure generation and propagation (Choi & Koh, 2008). Inflammation also exerts deleterious effects on glucose homeostasis, exacerbates oxidative stress, and secretion of pro‐inflammatory cytokines, which facilitate sarcopenia (Xu & Wen, 2023). Therefore, epileptogenesis and sarcopenia have a common cause, and patients with JME with severe epileptogenesis could have poor ASM response, which is also related to sarcopenia. A previous study revealed that a poor response to ASM in patients with epilepsy was associated with decreased mitochondrial pathology and function in skeletal muscle, consistent with our findings (Miles et al., 2012). Second, sarcopenia is related to asthenia, fatigue, impaired physical function, increased chemotherapy toxicity, impaired quality of life, and reduced survival (Bozzetti, 2017; Prado et al., 2016; Wheelwright et al., 2013). Chemotherapy frequently has a strong potential to cause severe toxicity, which may lead to dose delays, reductions, and even discontinuation of treatment, commonly known as dose‐limiting toxicities. The association between sarcopenia and poor tolerance to treatment has been observed in several studies and may originate from alterations in the distribution, metabolism, and clearance of drugs (Prado et al., 2009; Srdic et al., 2016). The cause of sarcopenia affecting the ASM response in patients with epilepsy is unclear; however, sarcopenia can affect pharmacokinetics by altering the volume of distribution of drugs (Ryan et al., 2019). Third, although patients with JME underwent MRI when newly diagnosed, we could not exclude the effects of seizure‐related muscle damage or ASM toxicity (Simm et al., 2017; Wooles et al., 2014). Frequent seizures can produce rapid skeletal muscle breakdown and sometimes result in life‐threatening rhabdomyolysis. Additionally, muscle trauma due to seizure attacks or ingestion of ASM can cause muscle damage. A previous study also demonstrated that patients taking ASM showed decreased lower limb muscle force compared to matched controls; this decrease may be attributed to the presence of sarcopenia in the former group (Simm et al., 2017).

This study had some limitations. First, this was a retrospective study conducted at a single tertiary hospital in which the TMT in patients with JME and healthy controls was measured using brain MRI. Further prospective studies with larger sample sizes conducted at multiple centers are needed to confirm our findings. Second, we manually measured the TMT, which could have caused a measurement bias. However, efforts were made to increase the homogeneity of this study by including only one experienced neuroradiologist. Third, patients with JME generally show good responsiveness to ASM. Therefore, we defined ASM poor responders as those not well controlled with the first ASM and were administered a second ASM. This definition was different from the definition of drug‐resistant epilepsy presented by the International League Against Epilepsy (ILAE), that is, failure of adequate trials of two tolerated and appropriately chosen and used ASM schedules (Kwan et al., 2010). Additionally, the response to ASM is often dynamic and can change over time (Brodie et al., 2012). However, our study is the first to investigate the relationship between ASM response and sarcopenia in patients with epilepsy.

## 5 CONCLUSION

We demonstrated that TMT did not differ between patients with JME and healthy controls but was reduced in ASM poor responders compared to ASM good responders, suggesting a link between ASM response and sarcopenia in patients with JME. In addition, we suggest that TMT can be used to investigate sarcopenia in various neurological disorders.

## AUTHOR CONTRIBUTIONS

*Conception and design*: Jinseung Kim, Ho‐Joon Lee and Kang Min Park. *Acquisition of data; analysis and interpretation of data*: Ho‐Joon Lee and Kang Min Park. *Drafting the manuscript or revising*: Jinseung Kim and Dong Ah Lee. *Final approval*: Kang Min Park.

## CONFLICT OF INTEREST STATEMENT

All authors have no conflicts of interest to declare at the time of submission.

### PEER REVIEW

The peer review history for this article is available at https://publons.com/publon/10.1002/brb3.3464.

## Data Availability

The data that support the findings of this study are available from the corresponding author upon reasonable request.
